# Clearance of Intracellular Pathogens with Hyaluronic Acid Nanomicelles Responsive to H_2_S and pH

**DOI:** 10.3390/molecules29245971

**Published:** 2024-12-18

**Authors:** Jun Luo, Hui Huang, Junfeng Jiang, Wenyu Zheng, Peng Chen, Hongjin Bai

**Affiliations:** Engineering Laboratory of Chemical Resources Utilization in South Xinjiang, Tarim University, Alar 843300, China; ljpaow@163.com (J.L.); 19590128436@163.com (H.H.); jikiry@163.com (J.J.); daisyzwy@163.com (W.Z.)

**Keywords:** hyaluronic acid, pH and H_2_S dual-responsive, intracellular bacteria, biofilm

## Abstract

Hyaluronic acid (HA) is an acidic mucopolysaccharide of animal origin composed of repeating disaccharide units of N-acetylglucosamine and glucuronic acid. Due to its excellent biocompatibility, biodegradability, and selective affinity for CD44 receptors on cell surfaces, HA is widely employed as a drug carrier. In our study, we aimed to target subcellular bacteria by grafting cystamine onto HA scaffolds through an amide reaction, producing a linker responsive to H_2_S and pH changes. Subsequently, hydrophobic dodecylamine was attached to HA, forming amphiphilic molecules. These amphiphilic entities can self-assemble into nanomicelles in an aqueous solution, thereby encapsulating the antibacterial agent triclosan (TCS). The resulting HA-based system (HASS-TCS) can be internalized via CD44-mediated endocytosis, releasing substantial amounts of streptomycin and TCS in H_2_S-rich and acidic environments. Additionally, HASS-TCS has demonstrated effectiveness in eradicating biofilms and addressing intracellular infections caused by *Salmonella*. This study underscores a novel pH-sensitive hyaluronic acid-based drug delivery system with significant potential for the effective treatment of intracellular infections.

## 1. Introduction

Surviving and proliferating in host cells, intracellular bacteria pose a significant challenge in infection treatment due to their ability to evade host immune responses and persist within phagolysosome killing mechanisms—even under harsh cellular conditions [1,2,3,4]. These pathogens utilize host cell defense mechanisms to shield themselves from antibiotic treatments, rendering them more difficult to eliminate than extracellular bacteria and posing a serious threat to human health [4]. Consequently, the effective eradication of intracellular bacteria remains a crucial focus in the field of bacterial infection therapy.

Clinically, antibiotics are the standard treatment for bacterial infections. However, challenges arise in treating intracellular infections because the presence of the host cell membrane and the complex intracellular environment impede effective antibiotic penetration, reducing intracellular antibiotic activity and accumulation [5,6,7]. Additionally, a mismatch between antibiotic and bacterial distribution within cells further diminishes the antimicrobial efficacy. As a result, intracellular bacteria can resist the bactericidal actions of most antibiotics, making single-drug treatments often insufficient to fully eradicate such infections [8,9,10].

Various synthetic and non-synthetic polymers are explored for their potential as carriers in drug delivery systems and as biodegradable materials [11,12,13,14]. Hyaluronic acid (HA) is a linear polysaccharide consisting of repeated disaccharide units comprising N-acetylglucosamine and glucuronic acid [15]. Hyaluronic acid is widely utilized as a kind of carrier for anti-tumor medications on account of excellent biocompatibility, biodegradability, and selectively binding to the CD44 molecule present on tumor cell surfaces [16,17,18]. Its distinctive biological properties, along with superior physicochemical attributes, have led to its significant use in both tissue engineering and drug delivery applications [19]. Ki Young Choi et al. synthesized colorless hyaluronic acid conjugates by chemically coupling hydrophobic 5β-cholic acid with the HA skeleton. Cellular experiments using Cy5.5-labeled HA nanoparticles demonstrated effective absorption by SCC7 cancer cells, which overexpress the HA receptor CD44 [20]. Yang X et al. encapsulated paclitaxel with HA to develop a nano drug carrier (HA-NLC). Compared with paclitaxel alone, these carrier materials exhibited better tolerance and anti-tumor activity [21]. Leveraging its excellent physical and chemical properties combined with extensive reference-based research, HA can be used to couple with various drugs. Utilizing the targeting properties of hyaluronic acid receptors, drugs can be concentrated within cells, making HA widely applicable in the field of drug delivery systems.

In our study, targeting the acid and H_2_S production characteristics of *Salmonella Typhimurium* during infection, streptomycin was grafted onto the hyaluronic acid backbone via an amide linkage to form an H_2_S- and pH-responsive linker, followed by the addition of dodecylamine to create amphiphilic molecules. The materials were characterized using high-performance liquid chromatography (HPLC), ^1^H NMR spectroscopy, scanning electron microscopy (SEM), zeta potential, and particle size analyses. We systematically investigated the release kinetics of HASS-TCS in pH and H_2_S environments and evaluated whether the mixture of TCS and streptomycin could enhance the efficiency in resisting intracellular bacteria. This study advances the understanding and strategic development of intracellular infection therapies by not only demonstrating the design concept of a dual-response system but also providing specific implementation methods to achieve the synergistic effects of antibiotics. It offers a novel experimental foundation and theoretical support for intracellular pathogen treatment, while also driving the further development of intelligent drug delivery systems.

## 2. Results

### 2.1. Synthesis and Identification of Micellar HASS

**Figure 1:**
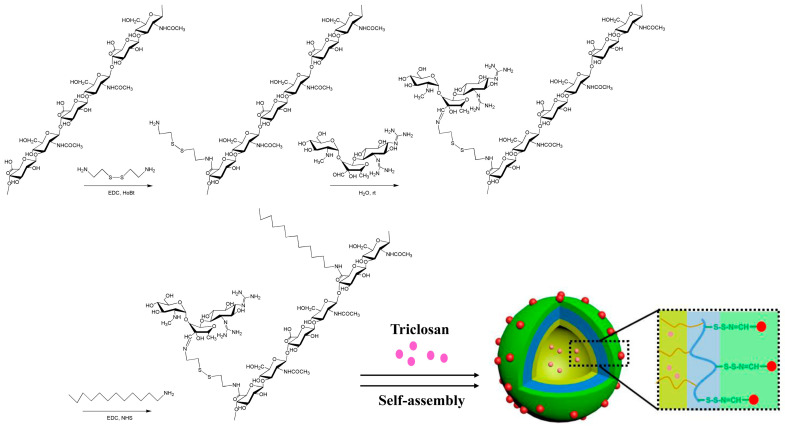
Schematic diagram of HASS synthesis route and loaded TCS.

**Figure 2:**
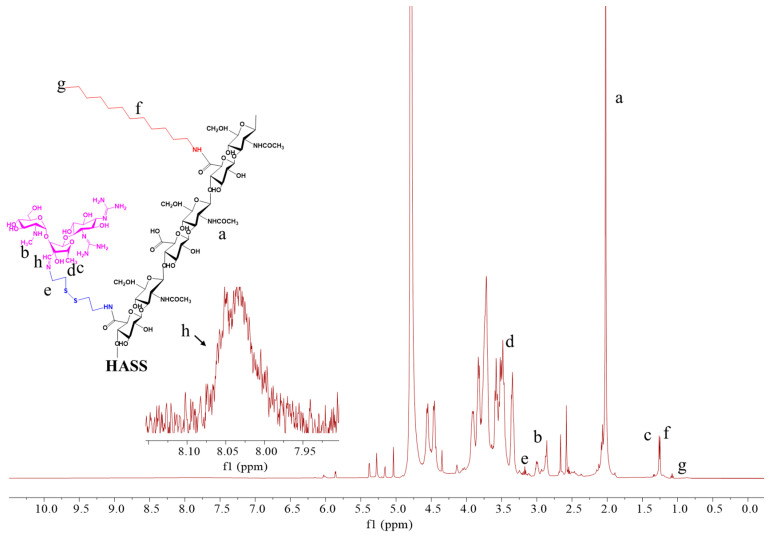
^1^H NMR spectra of blank HASS nanomicelles. Samples were dissolved in deuterium, and the spectrum was acquired at room temperature using a Bruker AM 500 spectrometer, a–h represent the positions of different H in the nuclear magnetic spectrum of the synthesized material.

The primary structure of empty HASS nanomicelles consists of two components: one is the reversible binding zone formed by the conjugation of cysteine to hyaluronic acid via acylhydrazone bonds with streptomycin, while the other is a hydrophobic unit composed of dodecylamine (Figure 1). According to the ^1^H NMR spectrum ratio of the methoxy proton substituted by Cys to the methoxy proton in HA, the degree of substitution of Cys in the HASS nanomicelles (the quantity of Cys every 100 disaccharide units within HA) was gauged to be 82. A comparison of the ^1^H NMR spectra of streptomycin and the synthesized complex reveals the absence of the aldehyde proton signal at 9.66 ppm in both spectra; however, a new signal appears at 8.03 ppm. This change indicates the formation of a hydrazone bond and confirms the successful coupling of streptomycin with the cysteine/hyaluronic acid complex (Figure 2 and Figure S1). A quantitative analysis of the guanidine groups yielded a streptomycin content of 8.7% (*w*/*w*). The extent of dodecylamine substitution was measured as three based on the integrated area proportion of the proton peak at 0.76 ppm aligning with the terminal methyl group of dodecylamine to the methyl proton peak of the acetyl group in hyaluronic acid in the ^1^H NMR spectrum of HASS. The molecular weight of HASS, as measured by HPGPC, was found to be 10.9 kDa (Figure S2).

### 2.2. Characterization of HASS and HASS-TCS Nanomicelles

**Figure 3:**
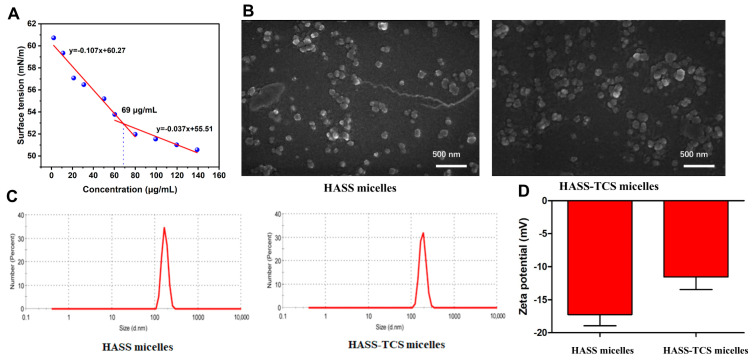
Characterization of both the blank HASS nanomicelles and HASS-TCS nanomicelles includes several key analyses. (**A**) The correlation between the blank HASS nanomicelles and surface tension at varying concentrations is examined. (**B**) Scanning electron microscopy (SEM) images illustrate the structure of the synthesized blank HASS nanomicelles alongside the drug-loaded HASS-TCS nanomicelles. (**C**) The average particle size distribution of both the blank HASS nanomicelles and drug-loaded HASS-TCS nanomicelles is assessed using dynamic light scattering (DLS). (**D**) The zeta potential measurements for the blank HASS nanomicelles and drug-loaded HASS-TCS nanomicelles are also reported.

The critical micelle concentration (CMC) of the HASS nanomicelles was gauged through surface interfacial tension measurements. Since the micelle concentration increased, the surface tension gradually decreased, reaching an inflection point at 69 μg/mL. This inflection point signifies a significant change in the behavior of the surfactant molecules in solution [22]. At the critical micelle concentration, amphiphilic molecules spontaneously self-assemble into micellar structures (Figure 3A). The CMC of blank HASS nanomicelles was found to be 69 μg/mL.

HASS-TCS nanomicelles containing 13.18% TCS were prepared, achieving an encapsulation efficiency of 78.54%. The self-assembly action of these nanomicelles within an aqueous solution was explored through scanning electron microscopy (SEM), dynamic light scattering (DLS), and zeta potential analysis. The particle sizes of both the HASS and HASS-TCS nanoparticles exhibited a relatively narrow range, conforming to a standard normal distribution, which indicates good dispersion, uniform particle size, and minimal aggregation. The average diameter of the nanomicelles was found to be 190 nm, aligning with the measurements obtained from the SEM. Notably, the volume of the drug-loaded HASS-TCS nanomicelles exceeded that of the HASS nanomicelles. These nanoparticles, approximately 200 nm in size, can evade recognition and capture by the reticuloendothelial system in vivo, facilitating passage through interstitial spaces and extending circulation time [23]. The zeta potential analysis revealed that both the HASS and HASS-TCS nanomicelles possess negative surface charges. The empty HASS micelle exhibited a potential of −17 mV, whereas the drug-loaded HASS-TCS micelle showed a potential of −11 mV. The higher zeta potential of the drug-loaded HASS-TCS micelle compared to the empty HASS micelle can be attributed to the negative charge of the carboxyl groups in hyaluronic acid.

### 2.3. The Effect of Different pH on the Release of Streptomycin and TCS

**Figure 4:**
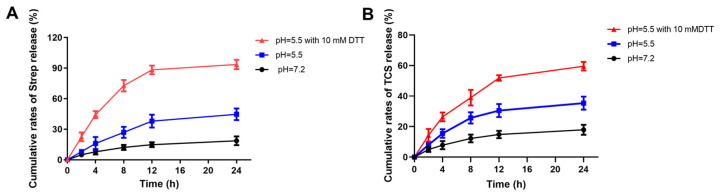
Release curves of Strep (**A**) and TCS (**B**) in HASS-TCS nanomicelles under different buffering environments.

At 12 h, 37.95% of streptomycin was released into an acidic medium with a pH of 5.5, while only 14.8% was effectively released from a neutral medium at pH 7.2. This significant difference is primarily due to the increased susceptibility of pH-sensitive hydrazone bonds to cleavage in acidic environments, leading to a substantial release of streptomycin into the medium. In our in vitro release experiments, we observed that the release of streptomycin stabilized at approximately 44.65% after 24 h. This phenomenon may result from the random grafting of cystine/Strep or deciamine onto hyaluronic acid, which allows the side chains to be distributed in both the internal and external regions of the nanomicelles. Furthermore, streptomycin may play a supportive role in the structure of the nanomicelles, consistent with the observed release of streptomycin in TCS (Figure 4) [24]. We simulated the production of H_2_S during the growth of *Salmonella Typhimurium* by adding DDT to the release medium. Upon analyzing the release medium, we found that the release of streptomycin and TCS in a buffer with pH 5.5 containing 10 mM DDT reached 93.15% and 59.58%, respectively, after 24 h. Notably, these release levels were significantly higher than those observed in the acidic medium at pH 5.5, indicating that the HASS-TCS materials exhibit dual responsiveness to pH and H_2_S.

### 2.4. Cell Toxicity Detection of HASS and HASS-TCS

**Figure 5:**
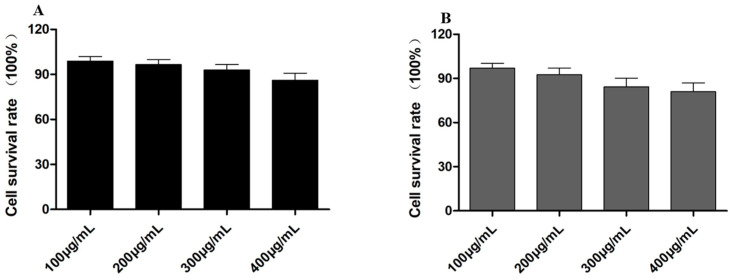
Cell viability of RAW264.7 cells cultured with blank HASS nanomicelles (**A**) (8.7% Strep) and HASS-TCS nanomicelles (**B**) (8.7% Strep, 13.2% TCS) of different concentrations for 48 h.

The MTT experiment was implemented to evaluate the cytotoxicity of the empty HASS nanomicelles and HASS-TCS nanomicelles loaded with TCS at concentrations between 100 μg/mL and 400 μg/mL. As illustrated in Figure 5, the results indicate that after co-incubation with RAW264.7 cells for 48 h, both the empty HASS nanomicelles and drug-loaded HASS-TCS nanomicelles exhibited a cell survival rate of approximately 90%, suggesting low cytotoxicity. Notably, the survival rate of the processed cells through the HASS nanomicelles loaded with TCS at a concentration of 300 μg/mL was lower than that of the control cells treated with the empty vector. This decrease may be attributed to the higher concentration of TCS in the nanomicelles, which results in the increased release of both TCS and streptomycin over the 48 h period, thereby affecting cell survival and demonstrating cytotoxic effects. Based on these findings, a concentration of 200 μg/mL was selected for all the subsequent experiments to evaluate intracellular activity.

### 2.5. HASS-TCS Effectively Removes Biofilms and Intracellular Bacteria

**Figure 6:**
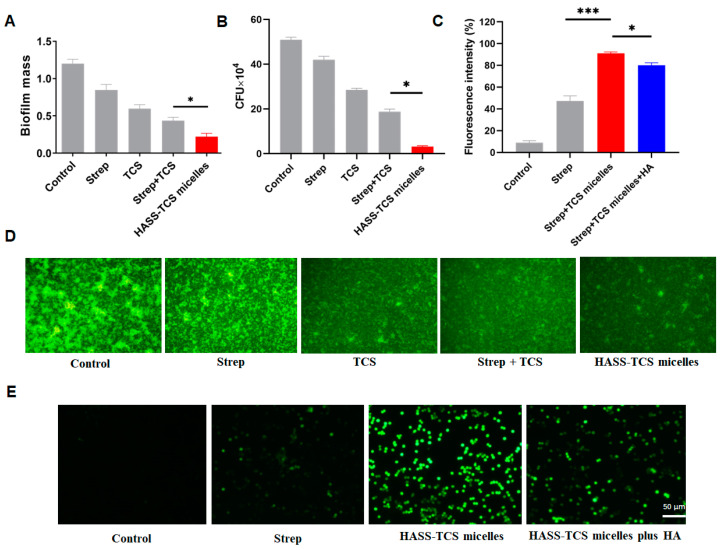
Functional assessment of the HASS-TCS nanomicelles: (**A**) Efficacy of the HASS-TCS nanomicelles in biofilm clearance. (**B**) The capability of HASS-TCS nanomicelles to eradicate intracellular bacteria. (**C**) Changes in fluorescence intensity within cells post-blockade of the CD44 receptor with hyaluronic acid (HA). (**D**) Fluorescence microscopy image demonstrating the disruption of *Salmonella Typhimurium* biofilms by the HASS-TCS nanomicelles. (**E**) Fluorescence microscopy images displaying the intracellular content of *Salmonella Typhimurium*. RAW264.7 cells infected with *Salmonella Typhimurium* were treated with HASS-TCS nanomicelles containing 8.7% streptomycin (Strep) and 13.2% triclosan (TCS), as well as equal concentrations of TCS, Strep, and their combination (Strep + TCS). Treatment was conducted at a micelle concentration of 200 µg/mL for 24 h. The presented data are averages from three independent experiments. “*” indicates (*p* < 0.05); “***” indicates (*p* < 0.001).

In the monotherapy group, both 200 µg/mL TCS and streptomycin effectively reduced the fluorescent signal associated with the biofilm of *Salmonella Typhimurium*. Notably, the combination of TCS and streptomycin demonstrated superior efficacy compared to either agent used alone. A significant difference was observed in the biofilm clearance capabilities between the group treated with the HASS-TCS nanomicelles at the same concentration and the mixture of TCS and Strep (Figure 6A,B). Following the treatment with HASS-TCS, there was a marked reduction in the biofilm mass of *Salmonella Typhimurium*, accompanied by a significant decrease in the aggregation of biofilm and bacteria. Some biofilm structures exhibited severe damage and had dispersed. Furthermore, fluorescence microscopy allowed for the visualization of individual bacteria, illustrating the impact of HASS-TCS on biofilm disruption. This evidence suggests that HASS-TCS is effective in degrading the biofilm structure of *Salmonella Typhimurium*.

Macrophages infected with intracellular bacteria (*Salmonella Typhimurium*) were incubated with 200 μg/mL of HASS-TCS (containing 8.7% streptococcus, 13.2% TCS) or equivalent amounts of TCS, Strep, and a simple mixture of both (Strep + TCS) for 12 h for intracellular viability counting. Both TCS and Strep could reduce the number of intracellular *Salmonella Typhimurium* wounds in the single dose group, and the efficacy of the simple mixture was better than that of the single dose, while there was a significant difference in the bactericidal ability of the HASS-TCS micelle group compared with the simple mixture. In other words, HASS-TCS nanomicelles have a stronger ability to scavenge intracellular bacteria (Figure 6B).

The limited ability of highly hydrophilic streptomycin to rapidly penetrate mammalian cell membranes hinders its effectiveness in eradicating all intracellular bacteria within a short timeframe [5,25]. Hyaluronic acid, serving as a natural ligand for the cell surface receptor CD44, can modulate immune responses through CD44-mediated endocytosis. To ascertain whether hyaluronic acid nanomicelles could enter cells via CD44 receptor-mediated endocytosis, we conducted experiments in which macrophages were pre-treated with free hyaluronic acid to block the receptor. Following this treatment, we measured the fluorescence intensity of streptomycin within the macrophages.

The quantification of intracellular streptomycin using immunofluorescence demonstrated that compared to the untreated group, the average fluorescence intensity in macrophages from the hyaluronic acid or streptomycin pre-incubation group was significantly diminished (Figure 6C,E). This finding strongly indicates that the blockade of CD44 receptors reduces the macrophage uptake of HASS-TCS nanomicelles, with their internalization attributed to CD44-mediated endocytosis. Consequently, HASS-TCS is internalized into cells via CD44-mediated endocytosis in the context of intracellular bacterial infection, thereby facilitating a series of antibacterial processes.

## 3. Discussion

In the early stages of intracellular bacterial infection, most pathogens remain within phagosomes and lysosomes, making the effective clearance of bacteria from lysosomes a crucial aspect of treating such infections [26]. Previous studies have reported that the acidic extracellular environment of lysosomes facilitates the widespread use of acid-sensitive hydrazone bonds based on glycosaminoglycans in drug delivery systems. Moreover, *Salmonella Typhimurium* produces H_2_S during its growth [27,28]. The overexpression of the CD44 receptor significantly enhances the endocytosis of hyaluronic acid-conjugated nanoparticles [29,30], and the acidic environment promotes the rapid degradation of micellar structures. Our research conjugated streptomycin to hyaluronic acids and dodecylamine-based amphiphilic molecules via hydrazone bonds, encapsulating TCS to form a pH-responsive and H_2_S-responsive antibacterial nanomaterial, HASS-TCS (Figure 1). The introduction of cystamine as a “bridge” for linking streptomycin and hyaluronic acid results in hyaluronic acid-based nanomicelles that not only rapidly release TCS and streptomycin under simulated acidic conditions but also exhibit significantly enhanced release rates in the presence of H_2_S gas in acidic environments. This suggests that HASS-TCS can more effectively and rapidly inhibit intracellular bacteria that produce H_2_S [24]. The HASS-TCS nanomicelle can enter cells via CD44 receptor-mediated macrophage endocytosis. The antibiotics and TCS released from the hyaluronic acid nanomaterial exhibit potent bactericidal effects against *Salmonella typhimurium*, significantly disrupting biofilm formation (Figure 6). Clearly, the combination of chemical antibacterial agents and antibiotics offers distinct advantages in eradicating intracellular bacteria, thereby effectively reducing the required doses of TCS and streptomycin.

Functionalized hyaluronic acid-derived nanoparticles and drug delivery systems present promising prospects for in vivo analysis and clinical applications [31]. Despite the dual responsiveness of HASS-TCS drug-loaded nanomicelles to pH and H_2_S, which enables the effective inhibition of intracellular bacteria and reduction in antibiotic dosage, their high cost and complex preparation process currently hinder clinical implementation. However, the system demonstrates significant potential for further optimization. By reducing costs and simplifying the preparation process, HASS-TCS nanomicelles may meet clinical requirements.

## 4. Materials and Methods

### 4.1. Materials

Hyaluronic acid (HA, Mw = 10 kDa) was supplied by Bloomage Freda Biopharm, Jinan Co., Ltd. (Jinan, China); 1-(3-Dimethylaminopropyl)-3-ethylcarbodiimide (EDC), and N-Hydroxysuccinimide (NHS), 1-hydroxybenzotriazole hydrate (HOBt hydrate), cystamine (Cys), dodecylamine (DDA) streptomycin (Strep), triclosan (TCS), and dithiothreitol (DDT) were purchased from McLink (Shanghai, China); and 3-methyladenine (3-MA) was purchased from Yuanye Biotechnology Co., Ltd. (Shanghai, China). RPMI1640 was purchased from Gibco (Grand Island, NY, USA) and FBS was supplied by Hyelone (Logan, UT, USA). MTT (3-(4,5-dimethylthiazol-2-yl)-2,5-diphenylaustralazole) was offered by Sigma, Inc. (St. Louis, MO, USA). The streptomycin enzyme-linked immunosorbent assay kit was purchased from enzyme-linked biotechnology Co., Ltd. (Shanghai, China).

### 4.2. Methods

#### 4.2.1. Coupling of Hyaluronic Acid and Cysteine

The method of linking cysteine with hyaluronic acid was slightly modified according to previous research [19,32]. In a round-bottom flask, 0.5 g of hyaluronic acid (HA) was lysed within 100 mL of deionized water. In this solution, 0.195 g of EDC and 0.169 g of HOBt were added, followed by magnetic stirring for 30 min. Afterward, 6.54 g of cystamine (Cys) was incorporated, and the reaction proceeded for 1 day before being terminated. The resulting mixture was delivered into one dialysis bag with a molecular weight cutoff of 3500 Da, where it was dialyzed against 0.1 M sodium chloride for 24 h. Subsequently, the dialysis was continued in deionized water for an additional 2 days to remove all unreacted materials. Finally, the product obtained from the dialysis was freeze-dried to yield a hyaluronic acid/cystamine complex.

#### 4.2.2. Coupling of Streptomycin with Hyaluronic Acid and Cysteine Complex

The formation of acid-sensitive bonds was achieved through a reaction between the aldehyde group on streptomycin and the amino group on cysteine, resulting in the formation of acylhydrazone bonds. The reaction proceeded as follows: We dissolved 200 mg of hyaluronic acid/cystamine complex in deionized water to prepare a solution of 10 mg/mL. We added 50 mg of streptomycin gradually to the reaction solution (pH = 6.2) and stirred it magnetically overnight. The next day, the reaction product was packed into a 3500 Da dialysis bag and dialyzed with deionization water for 1 day. Subsequently, freeze-drying yielded a complex connected to streptomycin.

#### 4.2.3. Synthesis of HASS

Fine-tuning was performed as described in Reference [33]; 100 mg of the above was dissolved in deionized water, and 65 mg EDC and 40 mg NHS were pre-stirred for half an hour, followed by 30 mg of dodecamine and stirred overnight (pH = 8.0). The next day, the reaction products were packed into 3500 Da dialysis bags and dialyzed with 0.1 M Nacl and deionized water for 1 day, respectively. In addition, the amphiphilic molecule products were named HASS after lyophilization.

#### 4.2.4. Preparation of HASS Nanomicelles Loaded with TCS

We loaded TCS into the HASS nanomicelles according to the method described in the literature [34]. We dissolved 10 mg of TCS in DMSO in advance to prepare a storage solution of 10 mg/mL. In a round-bottom flask, 30 mg of empty HASS nanomicelles were mixed with 10 mL of deionized water. A total of 300 µL of TCS was gradually added drop by drop to the solution of empty HASS nanomicelles, followed by uniform stirring for 12 h to facilitate the encapsulation of TCS within the carrier. Deionized water was dialyzed for 24 h to remove unencapsulated TCS and DMSO. The dialysis product was freeze-dried, and the sample at this time was the HASS-TCS nanomicelles loaded with TCS. The drug-loaded nanomicelles were sealed and stored in a refrigerator at 253 K for the subsequent experiments.

#### 4.2.5. Characterization of HASS-TCS Micellar Complex

We dissolved the complex containing HA, Strep, Cys, HA Cys, and coupling compounds related to Strep and HASS in 500 μL of D_2_O, and dissolved DDA in CDCl_3_ for the spectral analysis. The analysis was conducted using a Bruker Avance III 500 MHz NMR spectrometer (Bruker, Karlsruhe, Germany) at 298 K. The morphological assessments of the HASS and HASS-TCS were conducted using scanning electron microscopy (SEM, FEI APREO-S, Hillsboro, OR, USA) and dynamic light scattering (DLS, Malvern Instruments Ltd., Malvern, UK). For the SEM analysis, nanosuspensions prepared at 0.5 mg/mL were deposited (3 µL) onto cleaned silicon wafers and air-dried prior to imaging at 10 kV. The zeta potential and dimensions of both micelle formations were measured using a Malvern ZS90 analyzer. The drug loading of streptomycin within the HASS carriers was quantified by the ninhydrin assay, measuring absorbance at 540 nm against a pre-established standard curve to estimate the unencapsulated strep content [35]. The loading ability and encapsulation efficacy of TCS in nanomicelles were evaluated using high-performance liquid chromatography (HPLC, ALLIANCE E2695, Indianapolis, IN, USA). Acetonitrile served as the mobile stage. Moreover, a flow rate was designated to 1 mL/min and a detection wavelength was set to 227 nm [36]. In addition to the drug loading efficiency (DLE), the drug loading content (DLC) was figured out in light of the equations:

(1) $$\mathrm{Drug}\;\mathrm{loading}\;\mathrm{capacity}=W_{\mathrm{TCS}\;\mathrm{loaded}}/W_{\mathrm{HASS}}\times 100\%$$

(2) $$\mathrm{Drug}\;\mathrm{loading}\;\mathrm{efficiency}=W_{\mathrm{TCS}\;\mathrm{loaded}}/W_{\mathrm{TCS}\;\mathrm{added}}\times 100\%$$

#### 4.2.6. Critical Micelle Concentration

The key micelle concentration of the amphiphilic molecule HASS was determined using a surface interfacial tension meter. We prepared 5 mL of HASS micelle storage solution showing a concentration of 2 mg/mL using deionized water, and measured its surface tension value. Then, we diluted the solution in a certain proportion while measuring the surface tension value at that concentration. Each concentration sample was measured three times. We drew the surface tension change curve using the average value, with the surface tension value on the *y*-axis and the micelle density value on the *x*-axis.

#### 4.2.7. Drug Release of HASS-TCS Nanomicelles Loaded with TCS

To investigate the acid and H_2_S responsiveness of the HASS-TCS nanomicelles, we prepared buffer solutions of varying pH using PBS, supplemented with 50 mM DTT, as the release medium. Next, micelle solutions were created at a density of 2 mg/mL and placed into dialysis bags using a molecular weight cut-off of 3500 Da. These bags underwent dialysis against a PBS solution containing 1% Tween-80, maintained at 310 K and shaken at 100 rpm in a constant temperature shaker. The release of streptomycin in the medium was quantified using an ELISA kit, while the TCS levels were measured via HPLC. The cumulative release (CR) was figured out through the following formula:

(3) $$\mathrm{Cumulative}\;\mathrm{release}=\;(C_{t}\times 15+\sum C_{t-1}\times 3)/M_{0}\times 100\%$$

M_0_ indicates the total mass of the micelle drug, and C_t_ is the concentration of the released drug into the medium at time t.

#### 4.2.8. Cell Viability Assay

We assessed the cytotoxic effects of both empty carrier nanomicelles and drug-loaded nanomicelles on mouse macrophage RAW264.7 cells through the MTT experiment. First, we digested the adherent macrophages with trypsin, and then mixed them thoroughly. Next, we transferred 90 µL (about 8000 cells) of the cell suspension into one 96-well plate and cultured for 4 h to facilitate adhesion to the surface. We prepared samples at concentrations of 100, 200, and 300 μg/mL, ensuring three replicates for each concentration, with phosphate-buffered saline (PBS) serving as the control. We incubated the cultures for 24 h, after which we removed the medium and displaced it using a 0.5 mg/mL MTT solution. Following an additional 4 h incubation, we discarded the medium once more and added 100 μL of DMSO to dissolve the blue-purple formazan crystals formed within the cells. Finally, solution absorbance was measured at a wavelength of 570 nm through one enzyme-associated immunosorbent assay reader.

#### 4.2.9. Biofilm Removal Experiment

The concentration of *Salmonella Typhimurium* was adjusted to 10^8^ CFU/mL using TSB. Subsequently, 1 mL bacterial solution was introduced in each 24-well plate, with sterile PBS added around the edges. The biofilm was allowed to form by incubating the plate at 310 K for 24 h in a temperature-controlled incubator. Following this, the wells were mildly rinsed three times with 0.9% NaCl to eliminate planktonic bacteria from the culture medium. Afterward, 1 mL of fresh TSB medium was added along with 200 μg/mL of the HASS-TCS nanomicelles, as well as equal amounts of Strep, TCS, and a simple mixture of both, maintaining PBS as a positive control for 1 day. The culture was then aspirated, and the wells were rinsed thrice using PBS. The biofilm amount was quantified using crystal violet staining [37]. The procedure involved adding methanol for fixation for 15 min, and then removing the methanol. Next, 50 μL of a 0.1% crystal violet solution (prepared by lysing 0.1 g crystal violet within 100 mL deionized water) was applied for 30 min. After discarding the crystal violet solution, the wells were rinsed thrice using deionized water to eliminate excess dye. Subsequently, 33% (*v*/*v*) glacial acetic acid was supplemented with the intention of dissolving the crystal violet, and the mixture was shaken for 15 min. The absorbance was determined at 600 nm, with three replicates for each group. A higher absorbance indicated a greater biofilm quantity.

In the biofilm removal experiment, 1 mL of *Salmonella Typhimurium* (10^8^ CFU/mL) was introduced into a 24-well plate with cell culture inserts. The bacteria established a stable biofilm after being incubated at 310 K for 1 day. Then, the substrate was discarded, and the wells were gently rinsed thrice with PBS. Fresh TSB medium and 200 μg/mL of the HASS-TCS nanomicelles were added, along with equal amounts of Strep, TCS, and a simple mixture of both, with PBS serving as a positive control. The substrate was sucked out before thrice rinsing with PBS. The cells were immobilized using 4% paraformaldehyde for half an hour at room temperature (RT) and rinsed thrice using PBS. Then, 1 mL of 5-(4,6-dichlorotriazinyl) aminofluorescein (5-DTAF) at an ultimate concentration of 10 μg/mL [38] was nurtured at RT in the dark for 2 h, after which the wells were rinsed thrice with PBS, each rinse lasting 5 min. Finally, the tablets were mounted with an anti-fluorescence quencher and examined under fluorescence microscopy, capturing images for analysis.

#### 4.2.10. Anti Intracellular Bacterial Activity

As described in the reference [39], mouse macrophage RAW264.7 was inoculated into a 24-well plate using 1640 medium without bispecific antibodies and cultured until it covered about 80% of the plate. Different bacteria (*Salmonella Typhimurium*) were added in a ratio of infection multiplicity (MOI = 5) of 5. We placed the 24-well plate in a centrifuge at 1000 rpm/min for 5 min, then incubated it in a cell culture box for 1 h to allow bacteria to fully infect the cells. We discarded the culture medium, washed it three times with preheating, added 500 μL of 1640 substrate with 50 µg/mL gentamicin, and continued to culture for 1 h to kill extracellular planktonic bacteria. Then, the substrate was abandoned and rinsed thrice using PBS. Then, 200 µg/mL of HASS-TCS nanomicelles (equal amounts of Strep, TCS, and a simple mixture of Strep and TCS) were added to the non-dual antibody 1640 culture medium for 4 h. The substrate was removed and rinsed three times with PBS. A total of 500 μL containing 0.25% Triton X-100 was supplemented. Meanwhile, the cells were left at room temperature for 30 min to fully release the bacteria. After gradient dilution with PBS, the cells were coated on TSA plates and incubated at 310 K for 12 h in a constant temperature incubator to count colony-forming units (CFUs).

#### 4.2.11. CD44 Closed Experiment

In the CD44 blocking experiment involving infected cells, an initial treatment with 1 mg/mL of hyaluronic acid (molecular weight 10 KDa) was performed, followed by incubation in a cell culture box for 1 h. After this, various drugs were introduced and cultured for an additional 4 h. Afterward, the substrate was eliminated. In addition, the cells were rinsed twice using PBS. Then, they were immobilized through 4% paraformaldehyde for 15 min and rinsed thrice using PBS. To block nonspecific binding, 2% bovine serum albumin was applied for 1 h, after which the cells were washed thrice using PBS. Subsequently, they were incubated overnight at 277 K with 200 µL of rabbit anti-streptomycin monoclonal antibody (diluted 1:150). Following this, the cells were rinsed thrice using PBS, and 300 µL of a secondary antibody (Dylight 488-conjugated goat anti-rabbit IgG, diluted 1:200) was added. This mix was cultivated at RT in the dark for 2 h, followed by three rinses with PBS. Finally, the cover glass was carefully removed, it placed onto a slide, and sealed with an anti-fluorescence quenching agent. Observations and photographs were taken using a fluorescence microscope.

## 5. Conclusions

This research details the development and analysis of hyaluronic acid-based HASS-TCS nanomicelles. These nanomicelles can be internalized through the CD44 receptor, promoting the entry of Strep into host cells. Moreover, HASS-TCS shows a quick degradation in acidic environments and in the presence of H_2_S gas, resulting in a significant and swift release of Strep and TCS. In addition, HASS-TCS effectively eradicates biofilms and demonstrates strong inhibitory activity against intracellular bacteria. This study offers a promising approach for employing hyaluronic acid as a drug-transferring mechanism to treat intracellular infections.

## Data Availability

The datasets are provided by the corresponding author according to reasonable requirements.

## Supplementary Materials

The following supporting information can be downloaded at: https://www.mdpi.com/article/10.3390/molecules29245971/s1, Figure S1: ^1^H NMR spectra of HA (A), Strep (B), Cys (C), DDA (D), Cys/HA (E), Strep-Cys/HA (F) and HASS (G). Figure S2: HPGPC traces of HASS.

## References

[1] Mak T.W., Saunders M.E. (2005). The Immune Response: Basic and Clinical Principles.

[2] Finlay B.B., Cossart P. (1997). Exploitation of mammalian host cell functions by bacterial pathogens. Science.

[3] Laskay T., Van Zandbergen G., Solbach W. (2003). Neutrophil granulocytes–Trojan horses for Leishmania major and other intracellular microbes?. Trends Microbiol..

[4] Mitchell G., Chen C., Portnoy D.A., Siamon Gordon A. (2017). Strategies used by bacteria to grow in macrophages. Myeloid Cells in Health and Disease: A Synthesis.

[5] Farouk F., Azzazy H.M.E., Niessen W.M.A. (2015). Challenges in the determination of aminoglycoside antibiotics, a review. Anal. Chim. Acta.

[6] Palvannan T., Boopathy R. (2006). Interaction of aminoglycoside antibiotics with surface Asp and Glu residues of phosphatidylinositol-specific phospholipase C. Enzyme Microb. Technol..

[7] Weiss G., Schaible U.E. (2015). Macrophage defense mechanisms against intracellular bacteria. Immunol. Rev..

[8] Subramaniam S., Joyce P. (2021). Bioinspired drug delivery strategies for repurposing conventional antibiotics against intracellular infections. Adv. Drug Delivery Rev..

[9] Petit T.J.P., Lebreton A. (2022). Adaptations of intracellular bacteria to vacuolar or cytosolic niches. Trends Microbiol..

[10] Van Bambeke F., Michot J.M. (2003). Antibiotic efflux pumps in eukaryotic cells: Occurrence and impact on antibiotic cellular pharmacokinetics, pharmacodynamics and toxicodynamics. J. Antimicrob. Chemother..

[11] Bag B.G., Das S. (2017). Nanoarchitectures by hierarchical self-assembly of ursolic acid: Entrapment and release of fluorophores including anticancer drug doxorubicin. RSC Adv..

[12] Lu J., Hu J. (2011). A new dual-responsive organogel based on uracil-appended glycyrrhetinic acid. Org. Lett..

[13] Ji X., Guo J., Tian J., Ma K., Liu Y. (2023). Research progress on degradation methods and product properties of plant polysaccharides. J. Light Ind..

[14] Ji X., Cheng Y., Tian J., Zhang S., Jing Y., Shi M. (2021). Structural characterization of polysaccharide from jujube (*Ziziphus jujuba Mill.*) fruit. Chem. Biol. Technol. Agric..

[15] Alaniz L., Cabrera P.V. (2002). Interaction of CD44 with different forms of hyaluronic acid. Its role in adhesion and migration of tumor cells. Cell Commun. Adhes..

[16] Ossipov D.A. (2010). Nanostructured hyaluronic acid-based materials for active delivery to cancer. Expert Opin. Drug Delivery.

[17] Chen W.H., Luo G.F. (2017). Mesoporous silica-based versatile theranostic nanoplatform constructed by layer-by-layer assembly for excellent photodynamic/chemo therapy. Biomaterials.

[18] Cho H.J., Yoon H.Y. (2011). Self-assembled nanoparticles based on hyaluronic acid-ceramide (HA-CE) and Pluronic^®^ for tumor-targeted delivery of docetaxel. Biomaterials.

[19] Munar-Bestard M., Vargas-Alfredo N. (2024). Mangostanin hyaluronic acid hydrogel as an effective biocompatible alternative to chlorhexidine. Int. J. Biol. Macromol..

[20] Choi K.Y., Min K.H. (2009). Self-assembled hyaluronic acid nanoparticles as a potential drug carrier for cancer therapy: Synthesis, characterization, and in vivo bio-distribution. J. Mater. Chem..

[21] Yang X., Li Y. (2013). Hyaluronic acid-coated nanostructured lipid carriers for targeting paclitaxel to cancer. Cancer Lett..

[22] Li X., Ma L., Zhou Y., Lu X., Jing L., Jing D. (2024). Rheological behavior and solution pH response properties of nanoparticle-regulated low surface tension systems. J. Chem. Phys..

[23] Glavas L., Olsén P., Odelius K., Albertsson A.C. (2013). Achieving micelle control through core crystallinity. Biomacromolecules.

[24] Qiu Y., Lu C. (2019). Synergistic clearance of intracellular pathogens by hyaluronan-streptomycin nanomicelles encapsulated with rapamycin. Carbohydr. Polym..

[25] Kim D.Y., Sharma S.K. (2023). Development of novel peptide-modified silver nanoparticle-based rapid biosensors for detecting aminoglycoside antibiotics. J. Agric. Food Chem..

[26] McClure E.E., Chávez A.S.O. (2017). Engineering of obligate intracellular bacteria: Progress, challenges and paradigms. Nat. Rev. Microbiol..

[27] Li M., Sun J. (2021). Drug delivery systems based on CD44-targeted glycosaminoglycans for cancer therapy. Carbohydr. Polym..

[28] Liao J., Zheng H. (2018). Tumor-targeting and pH-responsive nanoparticles from hyaluronic acid for the enhanced delivery of doxorubicin. Int. J. Biol. Macromol..

[29] Abatangelo G., Vindigni V. (2020). Hyaluronic acid: Redefining its role. Cells.

[30] Stern R. (2004). Hyaluronan catabolism: A new metabolic pathway. Eur. J. Cell Biol..

[31] Vasvani S., Kulkarni P. (2020). Hyaluronic acid: A review on its biology, aspects of drug delivery, route of administrations and a special emphasis on its approved marketed products and recent clinical studies. Int. J. Biol. Macromol..

[32] Fu C., Li H. (2015). Conjugating an anticancer drug onto thiolated hyaluronic acid by acid liable hydrazone linkage for its gelation and dual stimuli-response release. Carbohydr. Polym..

[33] Vafaei S.Y., Esmaeili M. (2016). Self assembled hyaluronic acid nanoparticles as a potential carrier for targeting the inflamed intestinal mucosa. Carbohydr. Polym..

[34] Tyrrell Z.L., Shen Y. (2010). Fabrication of micellar nanoparticles for drug delivery through the self-assembly of block copolymers. Prog. Polym. Sci..

[35] Pretorius D.C., Van Staden J.F., Botha A.D.P. (1991). An automated colorimetric method for the determination of cyanoguanidine in water. Water SA.

[36] Peris-Camarasa B., Dualde P. (2024). Fast and eco-friendly analytical method to determine bisphenols, parabens, benzophenone-3 and triclosan in human urine by ultra-performance liquid chromatography coupled to mass spectrometry. Microchem. J..

[37] Sun B., Sun M. (2024). Magnetic hydrogel micromachines with active release of antibacterial agent for biofilm eradication. Adv. Intell. Syst..

[38] Ferriol-González C., Domingo-Calap P. (2020). Phages for biofilm removal. Antibiotics.

[39] Song M., Wang J. (2024). Isolation, structural characterization and immunomodulatory activity on RAW264. 7 cells of a novel exopolysaccharide of Dictyophora rubrovalvata. Int. J. Biol. Macromol..

