# Supplementary material for: Clearance of Intracellular Pathogens with Hyaluronic Acid Nanomicelles Responsive to H2S and pH
Source: Molecules. 2024 Dec 18;29(24):5971. doi: 10.3390/molecules29245971 (PMC11678907; doi:10.3390/molecules29245971)
Supplement: Supplementary file 1 [file molecules-29-05971-s001.zip › molecules-3329686-supplementary/Supporting Information.pdf]

Clearance of Intracellular Pathogens with Hyaluronic Acid Nanomicelles Responsive to H_2S and pH

Jun Luo, Hui Huang, Junfeng Jiang, Wenyu Zheng, Peng Chen* and Hongjin Bai *

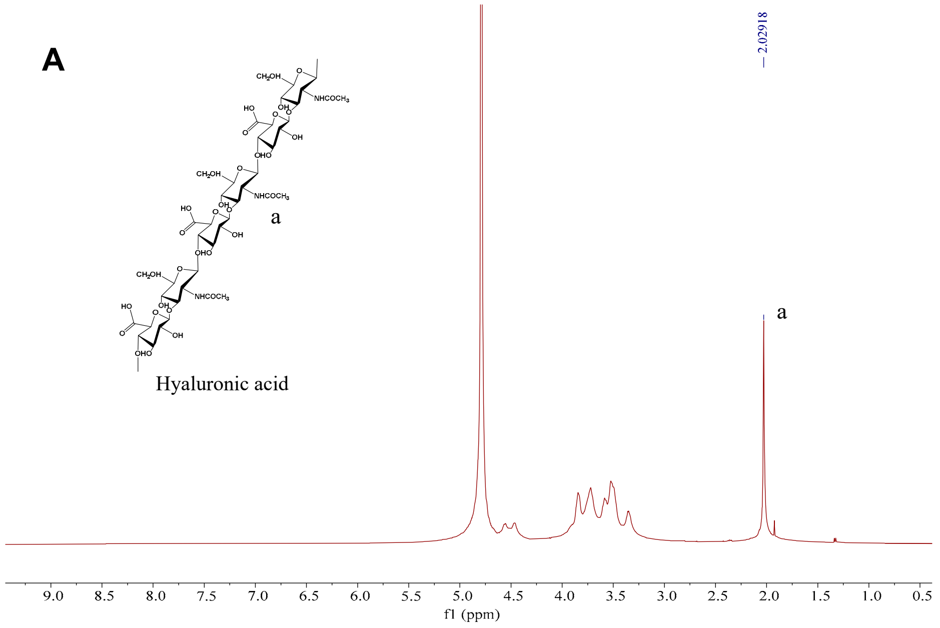

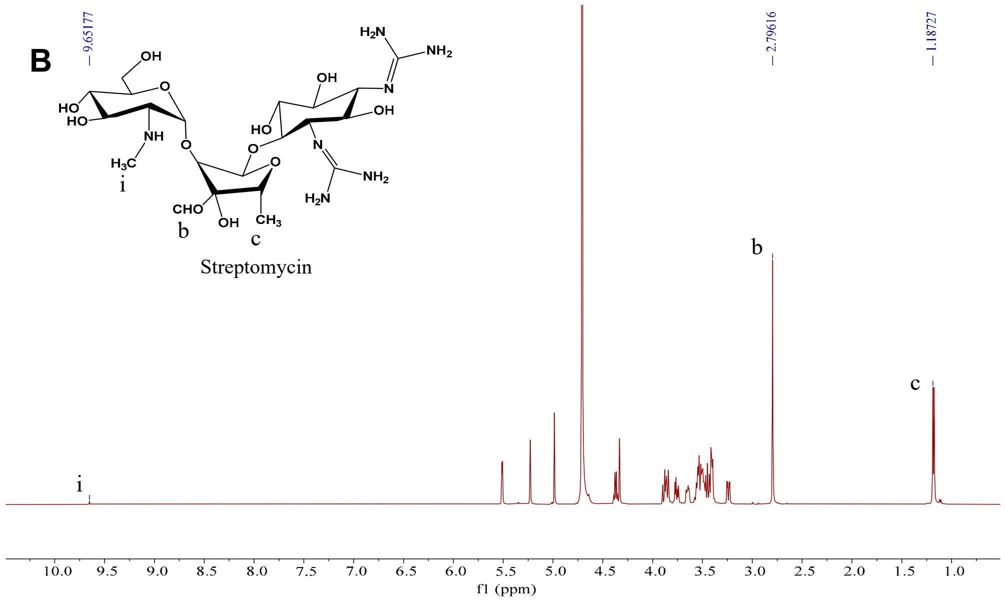

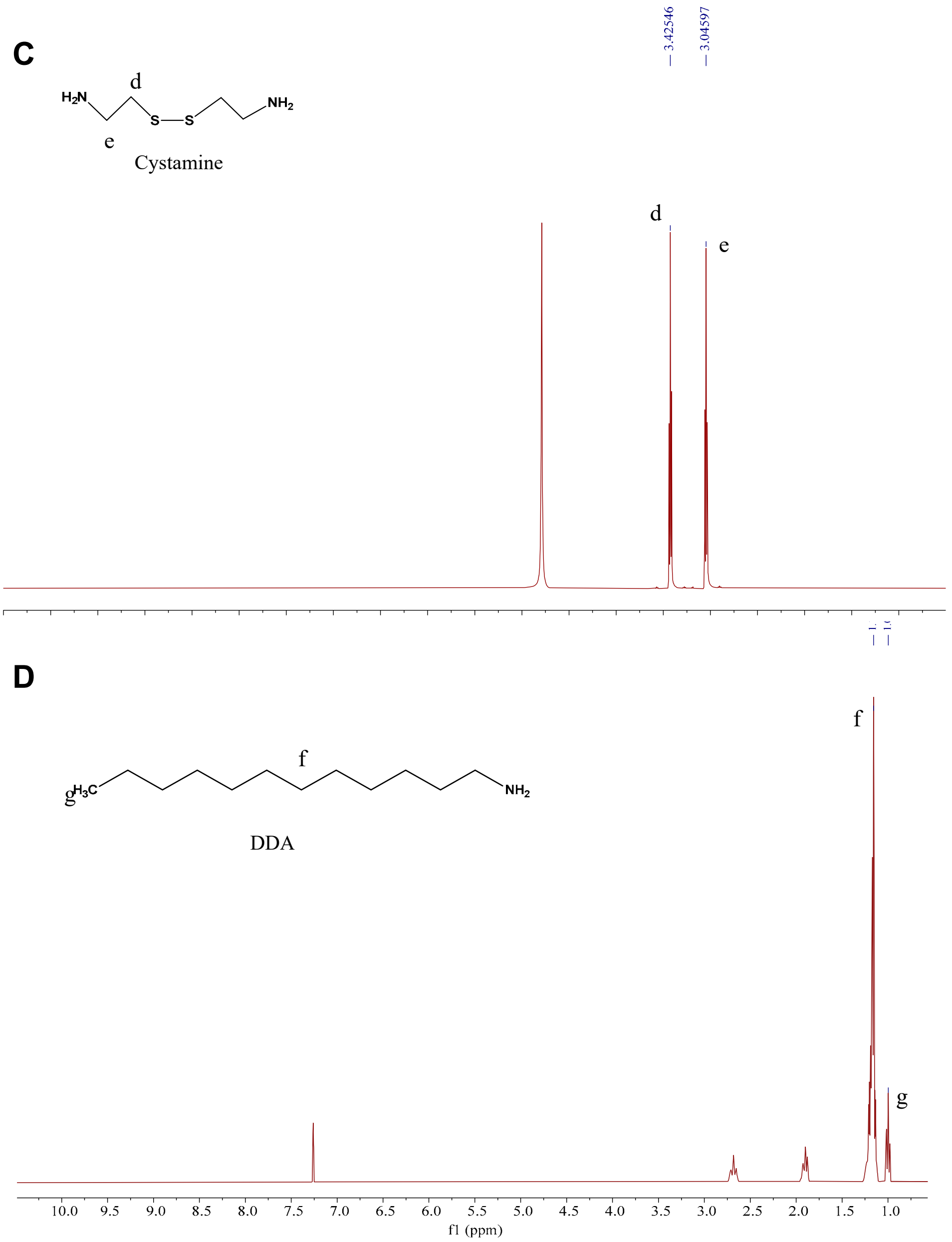
C
d
H2N
S—S
NH2
e
Cystamine
3.42546
3.04597
d
e
D
f
g H3C
NH2
DDA
f
g
10.0 9.5 9.0 8.5 8.0 7.5 7.0 6.5 6.0 5.5 5.0 4.5 4.0 3.5 3.0 2.5 2.0 1.5 1.0
f1 (ppm)

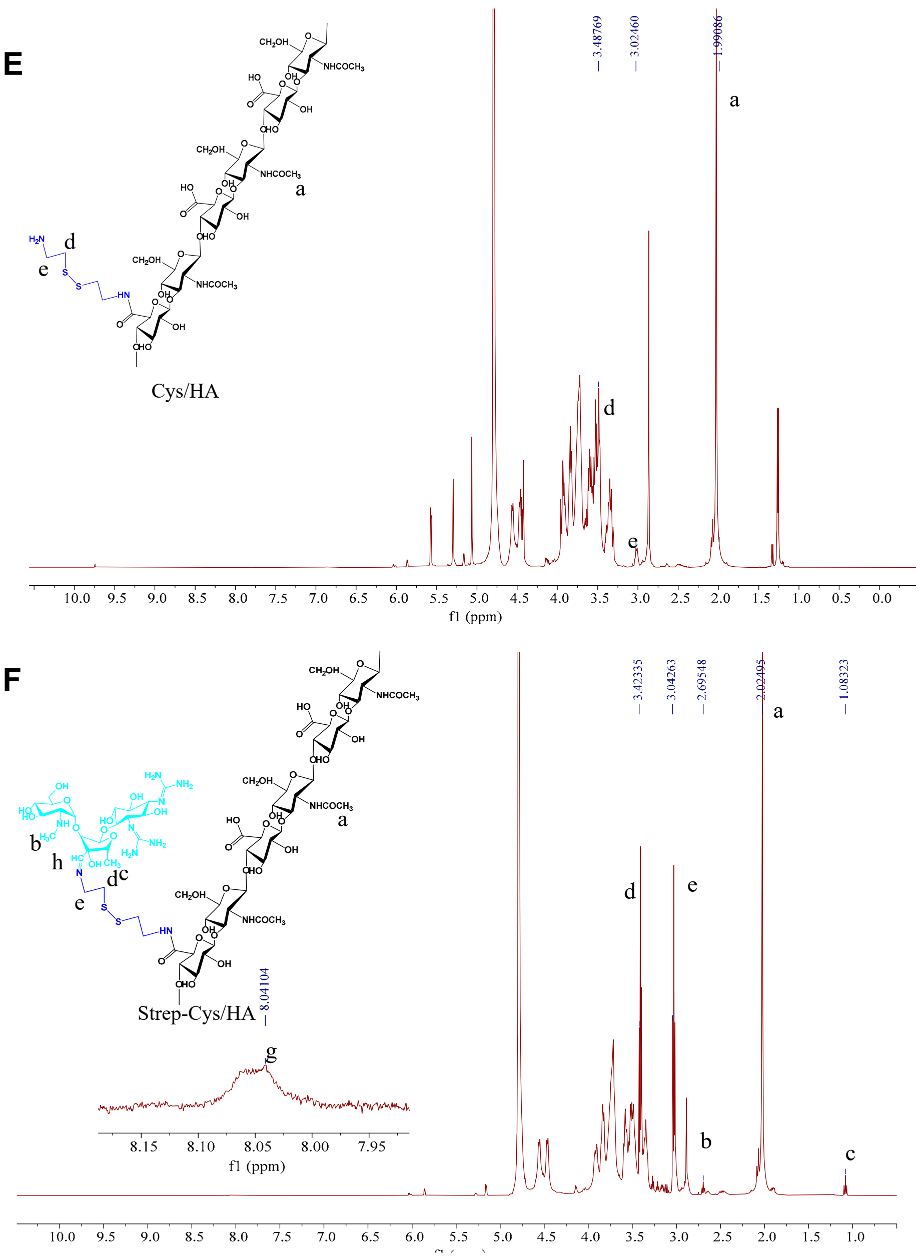
E
Cys/HA
a
d
e
3.48769
3.02460
1.99086
10.0 9.5 9.0 8.5 8.0 7.5 7.0 6.5 6.0 5.5 5.0 4.5 4.0 3.5 3.0 2.5 2.0 1.5 1.0 0.5 0.0
f1 (ppm)
F
Strep-Cys/HA
a
b
h
dc
e
d
e
g
b
c
3.42335
3.04263
2.69548
2.02495
1.08323
8.04104
8.15 8.10 8.05 8.00 7.95
f1 (ppm)
10.0 9.5 9.0 8.5 8.0 7.5 7.0 6.5 6.0 5.5 5.0 4.5 4.0 3.5 3.0 2.5 2.0 1.5 1.0

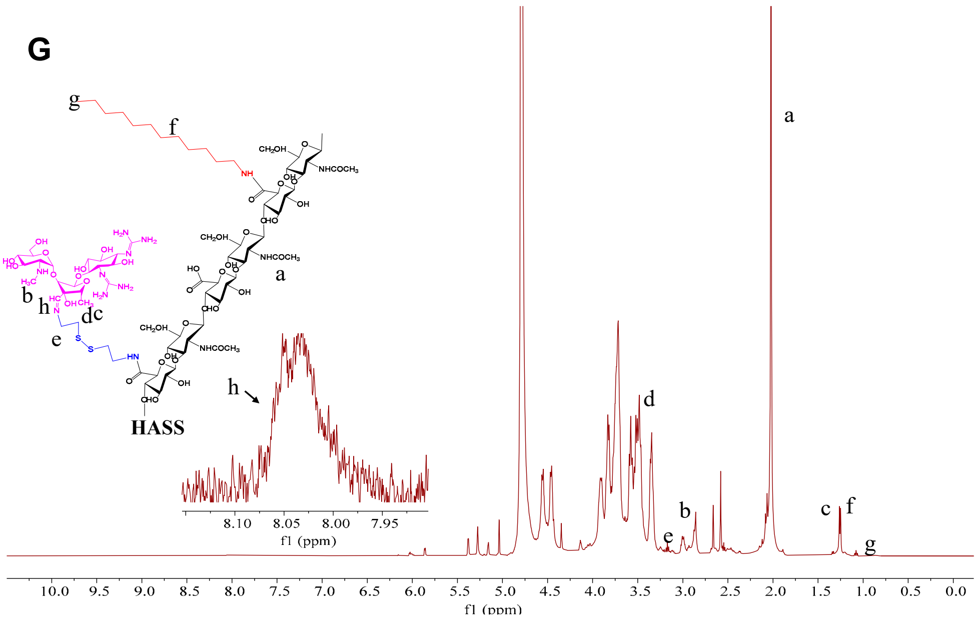

Figure. S1 ^{1}H NMR spectra of HA (A), Strep (B), Cys (C), DDA (D), Cys/HA (E), Strep-Cys/HA (F) and HASS (G).

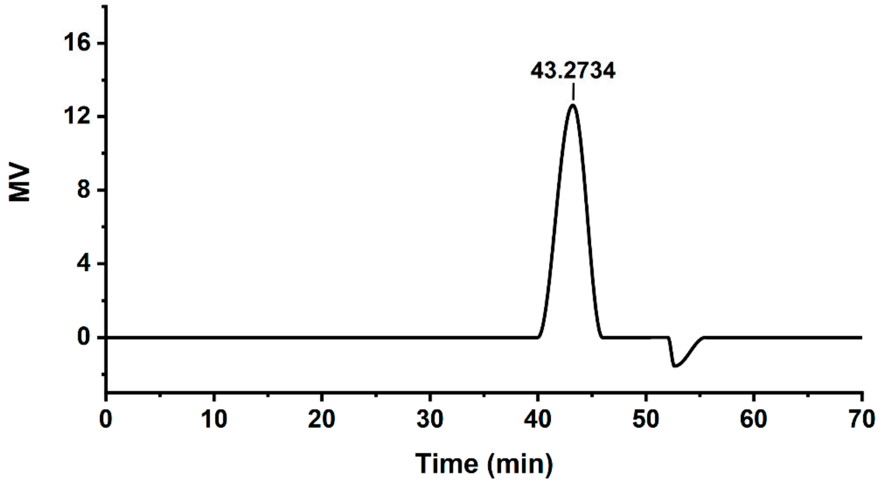

Figure. S2 HPGPC traces of HASS.
